# Dangerous Dysrhythmias in the ED: An Interactive EKG Workshop for Emergency Medicine Clerkship Learners

**DOI:** 10.5070/M5.52195

**Published:** 2026-07-31

**Authors:** Jordan Ancmon, Jacob Feldman

**Affiliations:** * The University of Texas Health Science Center at San Antonio, Department of Emergency Medicine, San Antonio, TX

## Abstract

**Audience:**

This guided small group exercise is suitable for medical students who have completed basic lectures on electrocardiogram (EKG) interpretation. This activity was created for use within a third year required Emergency Medicine (EM) clerkship but can be implemented in other undergraduate medical education settings as well.

**Introduction:**

Electrocardiogram (EKG) interpretation is poorly understood by medical students,^1^ and trainees display a concordant lack of confidence in their interpretation skills which can carry into fourth year of medical school and residency.^2^,^3^,^4^ Improving learner confidence and competence in identification of arrhythmias is of paramount importance to improve patient care.^5^,^6^ Our workshop targets the recognition and management of dangerous arrhythmias frequently seen in the Emergency Department

**Educational Objectives:**

By the end of this session, learners will be able to: 1) identify common tachyarrhythmias and pulseless rhythms using EKGs, 2) differentiate stable from unstable tachyarrhythmias based on hemodynamics, symptoms, and EKG findings in acutely ill ED patients, 3) apply appropriate critical resuscitative actions to manage stable tachyarrhythmias, unstable tachyarrhythmias, and pulseless rhythms during emergency department resuscitations, in accordance with ACLS (Advanced Cardiopulmonary Life Support) principles, and 4) construct a rapid, ED-focused decision-making algorithm to guide the identification and management of tachyarrhythmias and pulseless rhythms.

**Educational Methods:**

Learners participated in an interactive EKG workshop interspersed with lectures, EKG interpretation exercises, and case-based learning activities. During the workshop, students constructed a tachyarrhythmia and pulseless algorithm that will enable students in the future to identify and manage sinus tachycardia, supraventricular tachycardia (SVT), atrial fibrillation ( A fib ), atrial flutter, ventricular fibrillation (VF) and ventricular tachycardia (VT). Students collaborated in small groups, receiving real-time feedback from the facilitator on all activities.

**Research Methods:**

Since this curriculum was designed for research purposes, learners completed a pre-test prior to starting this session and a post-test once the session concluded. This EKG pre/post-test was developed alongside the workshop curriculum to address the concerns identified in the needs assessment. The test was validated through testing on PGY1-3 EM residents. Confidence was assessed using Likert-scales of 1 (not at all confident) to 5 (extremely confident). Retention was assessed four weeks after completing the workshop using the same post-test. This study was Institutional Review Board (IRB) exempt.

**Results:**

Pre and post-test results were analyzed for 69 students that were divided across four cohorts. Students averaged a pre-test score of 74% and post-test score of 87.75%. This post-test score was maintained at the four-week reassessment with an average score of 86% (24.6% response rate).

Confidence was measured by defining Likert scores of 1–3 as being “not confident” and 4–5 as “confident.” On the pre-test, student confidence in identifying ventricular tachycardia was 52%, identifying a shockable rhythm 38%, and identifying features of instability 16%. On the post-test, student’s confidence in identifying ventricular tachycardia rose to 96%, shockable rhythms to 93%, and features of instability to 90%.

**Discussion:**

Active small-group EKG workshops enhance learners’ confidence and competence in recognizing and managing clinically significant arrhythmias in the emergency department. This learning exercise has become a staple of our required third-year EM clerkship and continues to show high learner satisfaction.

**Topics:**

EKG, arrythmias, tachyarrhythmia, atrial fibrillation, ventricular tachycardia, PEA, asystole, ventricular fibrillation, atrial flutter, supraventricular tachycardia, undergraduate medical education, learner confidence, learner competence.

## USER GUIDE

**Table:** 

List of Resources:	
Abstract	1
User Guide	3
Small Groups Learning Materials	7
Appendix A: Activity A	11
Appendix B: Activity B	16
Appendix C: Activity C	21
Appendix D: Activity D	23
Appendix E: Learner Algorithm Tachyarrhythmia Blank	24
Appendix F: Learner Algorithm Tachyarrhythmia Key	25
Appendix G: Learner Algorithm pulseless Blank	26
Appendix H: Learner algorithm pulseless Key	27
Appendix I: Pre- & Post-Test	28
Appendix J: Pre- & Post-Test Key	35
Appendix K: Case-Based Learning Exercises	42
Appendix L: Case-Based Learning Exercises Key	55
Appendix M: PowerPoint	68

**Learner Audience:**

Third-year undergraduate medical students enrolled in the Emergency Medicine clerkship.

**Time Required for Implementation:**

**Table:** 

Activity	Learner time allotted	Answer review time
Pre-test	10 minutes	Not reviewed
Activity A, B, C	5 minutes per activity	5 minutes per activity
Break	10 minutes	NA
Activity D	5 minutes	5–10 minutes
Case-based Learning Exercises	10 minutes each case= 30 minutes total	10–20 minutes
Post-test	10 minutes	Not reviewed


**Recommended Number of Learners Per Instructor:**

20 students, divided into 4 or 5 groups

**Topics:**

EKG, arrythmias, tachyarrhythmia, atrial fibrillation, ventricular tachycardia, PEA, asystole, ventricular fibrillation, atrial flutter, supraventricular tachycardia, undergraduate medical education, learner confidence, learner competence.

**Objectives:**

By the end of this session, learners will be able to:

1. **Identify** common tachyarrhythmias and pulseless rhythms using EKGs.
2. **Differentiate** stable from unstable tachyarrhythmias based on hemodynamics, symptoms, and EKG findings in acutely ill ED patients.
3. **Apply** appropriate critical resuscitative actions to manage stable tachyarrhythmias, unstable tachyarrhythmias, and pulseless rhythms during emergency department resuscitations, in accordance with ACLS principles.
4. **Construct** a rapid, ED-focused decision-making algorithm to guide the identification and management of tachyarrhythmias and pulseless rhythms in the ER.

### Linked objectives and methods

This activity was created following the Dick and Carey model of instructional design^7^ through performing a learner-needs assessment in which a convenience sample of 15 students rotating on the EM clerkship were surveyed using Google JamBoard, and we identified weaknesses in interpretation of tachyarrhythmias and pulseless rhythms. We used these weaknesses to outline our specific learning objectives, and then using these learning objectives and specific weaknesses, we created a *de novo* pre- and post-test to measure learner outcomes which was validated and revised through testing on PGY1-3 EM residents.

In our literature review, we identified that others have attempted to create educational activities to meet this need previously. Some have instituted longitudinal weekly case-based EKG activities following basic EKG education,^8^ while others have attempted to use longitudinal web-based modules.^9^ Although no ideal learning modality has been identified, a lecture and workshop hybrid has shown to be effective compared to self-study.^1^ We chose the Emergency Medicine clerkship given the relative frequency with which these emergencies are treated in the clinical environment.

Thus, we identified a learning need important to future clinical performance, developed learning objectives and a specific tool to address these needs, and performed a literature review to identify a small-group teaching modality that would adequately fit our needs within the EM third-year clerkship. Because much existing literature delving into instructional methods evaluates learner confidence, we sought to test this, as well as expanding to determine improvement in learner competence by using multiple-choice testing on knowledge content.

### Recommended pre-reading for facilitator

Instructors should have baseline competence in interpreting Advanced Cardiac Life Support (ACLS) algorithms and EKG features identifying and differentiating sinus tachycardia, supraventricular tachycardias (SVT), atrial fibrillation (A Fib), atrial flutter, ventricular tachycardia (VT), and ventricular fibrillation (VF).

Review slides prior to the activity. All answers to Google Form activities and algorithms are within the power point slides (Appendix M).

### Small group application exercise (sGAE)

See the following attached materials for this small group exercise

- Appendix A: Activity A
- Appendix B: Activity B
- Appendix C: Activity C
- Appendix D: Activity D
- Appendix E: Learner Algorithm Tachyarrhythmia Blank
- Appendix F: Learner Algorithm Tachyarrhythmia Key
- Appendix G: Learner Algorithm pulseless Blank
- Appendix H: Learner algorithm pulseless Key
- Appendix I: Pre- & Post-Test
- Appendix J: Pre- & Post-Test Key
- Appendix K: Case-Based Learning Exercises
- Appendix L: Case-Based Learning Exercises Key
- Appendix M: PowerPoint

### Results and Tips for Successful Implementation

Learners were given a pre-test to complete prior to beginning the small-group activity and a post-test upon conclusion. Sixty-nine learners completed the 29 question pre- and post-tests across four EM clerkship blocks. To assess confidence and competence on the same exam, the pre/post-test would ask learners to identify important features of EKG interpretation (eg, rate, rhythm, and intervals), appropriate management for these arrhythmias, and how that management would change based on whether the patient was stable or unstable or had a pulse or was pulseless. These questions also asked learners how to determine if a patient was stable or unstable. For each of these multiple-choice questions, we also included Likert-type questions ranking from 1–5 their confidence in their answers, with a rank of 1 being defined as not at all confident to 5 being defined as extremely confident. This allowed us to determine if learners were confident in correct answers, or if they had guessed correctly, or if they were confident in incorrect answers.

Our study was adequately powered to detect a difference between pre- and post-testing. We calculated that to detect a difference of 5% between pre- and post-testing, we needed 34 participants to achieve a power of 80%, assuming a standard deviation of 10.

For determination of learner competence, we graded the percent of answers that were correct with the pre-test average for all students being 74% and the post-test average for all students being 87.75% (see tables 1). One month following the workshop, students were sent the same post-test again to test for retention. The 17 respondents (24.6% response rate) scored generally the same with a mean of 86%.

Confidence was assessed using Likert-scale data, which was averaged and analyzed as 1–3= not confident/disagree and 4–5= confident/strongly agree with the provided statement. Forty-three percent of all participants rated their confidence in the management of “shockable” versus “unshockable” rhythms as strongly confident on the pre-test compared to 94.9% on the post-test. Ninety-four-point four percent of all participants agreed that the workshop increased their confidence in the management of pulseless rhythms. An average of all questions across all four cohorts demonstrates an increase of confidence in student answers from 33.1% on the pre-test to 90.3% on the post-test. This increase in confidence was maintained on the second post-test taken one month following the EKG workshop at 87.8%.

Learner feedback was obtained through verbal discussion and was largely positive. They enjoyed the opportunity to review this initially challenging material so they could feel more confident in their management of patients with arrhythmias during their residency. Learners also enjoyed the interactivity of this activity as it provided a way to share didactic knowledge in lecture format briefly before letting learners play with the learning concepts themselves. Groups were randomly formed which we hoped would enable peer-to-peer teaching. This small group format of EKG interpretation has trickled down into the basic science curriculum, and now our medical school uses a similar format for teaching basics of EKG interpretation and arrhythmia identification.

For successful implementation we suggest that even during the lecture portion that learners be polled and invited to participate in discussion as much as possible. During small-group breakouts the instructor should be readily available to answer questions and to share important insights with the larger group as a whole as they’re discovered. The instructor will need to recognize that different groups will have different strengths and weaknesses in EKG interpretation, and so the instructor should try to discover each group’s abilities and teach to these specifically to maximize learner engagement and improvement. Additionally, we recommend eliminating the pre- and post-test if not planning to collect data. Facilitators can select specific components of the workshop to best fit their time and learners’ needs.

### Pearls

Learning pearls are captured in the algorithm handout that learners are provided at the beginning of the session (Appendices E and G).

## Supplementary Information











## Figures and Tables

**Figure:**
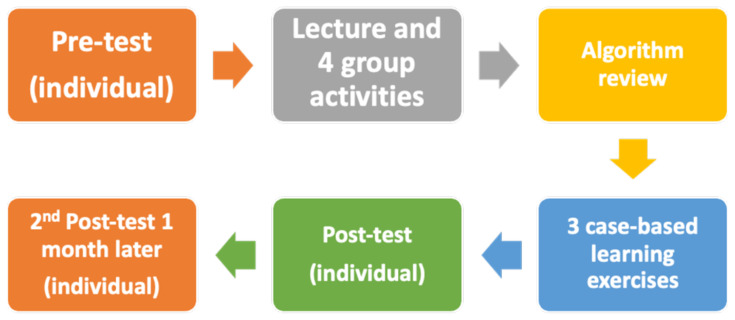


**Figure:**
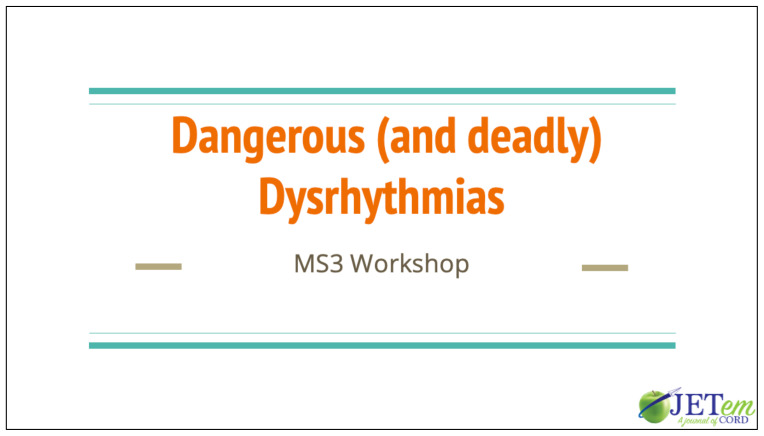
Please see associated PowerPoint file

## SMALL GROUPS LEARNING MATERIALS

### Small Group Application Exercise (sGAE)

#### 1. Group formation

Count of participants one by one to form groups of 3–4 students.

Pre-test Allow participants 10 minutes to complete the pre-test (Appendix I) individually.

#### 2. Identification and Classification of Tachyarrhythmias

Following the pre-test, the facilitator will give a short PowerPoint presentation (Appendix M) on tachyarrhythmias and classification using a paradigm of supraventricular versus ventricular arrhythmias. Groups will then complete Activity A (Appendix A) interpreting the rhythm and rate of 5 EKGs with the following answers:

1. Sinus tachycardia
2. Atrial flutter
3. SVT (supraventricular tachycardia)
4. Atrial fibrillation with RVR (rapid ventricular response)
5. Ventricular tachycardia.

The facilitator will then review the answers to activity A via PowerPoint. Again, a short PowerPoint presentation will be reviewed on QT interval and classification using narrow versus wide. Groups will then complete activity B (Appendix B) interpreting the rhythm and QRS duration as narrow or wide for 5 EKGs. Again, the facilitator will review the answers via PowerPoint.

#### 3. Identification and Classification of Pulseless Rhythms

The instructor will review using ACLS the four pulseless rhythms and which can be defibrillated. The groups will then complete activity C ( Appendix C) interpreting the pulseless rhythm and selecting if the rhythm could be defibrillated. The facilitator will review the answers via PowerPoint.

The facilitator will review the reversible causes of asystole and PEA (pulseless electrical activity) described by the 5 H’s and 5 T’s and their respective interventions. The groups will then complete activity D (Appendix D) with vignettes requiring participants to select the most likely H or T causing a non-shockable rhythm and the appropriate treatment. The answers will then be reviewed via PowerPoint. Participants will then take a 10-minute break following activity D.

#### 4. Management of Tachyarrhythmias and Pulseless rhythms

The facilitator will review the treatments for pulseless ventricular tachycardia and ventricular fibrillation as well as indications for cardioversion, defibrillation and transcutaneous pacing.

Participants will be given a framework for how to classify unstable versus stable tachyarrhythmias and their treatments. The answers to both algorithms will then be reviewed via PowerPoint and groups will be instructed to complete the 3 case-based learning exercises in their groups (Appendix L).

#### 5. Cases

Participants will be allowed to use algorithms during the case-based learning exercises. Each case features a patient vignette, 2 EKGs, and free text questions on identification, workup, and management. Cases include an inferior STEMI (ST Elevation Myocardial Infarction) who progresses into pulseless ventricular tachycardia, a new atrial fibrillation with RVR who requires cardioversion, and an SVT who following cardioversion progresses into ventricular fibrillation. Cases are to be completed in groups. The groups will then share their answers with the cohort, and the facilitator should provide immediate feedback on answers using the provided answer key.

#### 6. Post-test

Each individual student will then be instructed to take the post-test (Appendix I) and encouraged to utilize the algorithm during the post-test. Post-test questions are identical to pre-test questions with the addition of questions on the perceived effectiveness of the workshop. All participants will be sent the same post-test via email one month later.

#### Appendices

Appendix A: Activity A

Appendix B: Activity B

Appendix C: Activity C

Appendix D: Activity D

Appendix E: Learner Algorithm Tachyarrhythmia Blank

Appendix F: Learner Algorithm Tachyarrhythmia Key

Appendix G: Learner Algorithm Pulseless Blank

Appendix H: Learner algorithm Pulseless Key

Appendix I: Pre- & Post-Test

Appendix J: Pre- & Post-Test Key

Appendix K: Case-Based Learning Exercises

Appendix L: Case-Based Learning Exercises Key

Appendix M: PowerPoint

##### Instructor Guide

The instructor will need to print a packet containing the two blank algorithms (Tachyarrhythmia and Pulseless) (Appendix E and G) and 3 three case-based learning exercises (Appendix L). The formatting and printing of cases is important because turning the page reveals the patient’s subsequent clinical course.

Print handouts

Tachyarrhythmia algorithm (Appendix E)
Pulseless algorithm (Appendix G)
Case-based learning exercises (Appendix L)

Answer Keys

Tachyarrhythmia (Appendix F)
Pulseless (Appendix H)
Case-based learning (Appendix M)

##### Activity Structure

1. Have students take the pre-test individually (10 minutes) at the beginning of the workshop (Appendix I).
2. Pass out packets.
3. Randomly divide learners into equal-sized groups, ideally 3–4 learners per small group.
4. Present and work through the PowerPoint (Appendix M), encouraging learner involvement.
5. When prompted by slides, have students work through each activity in their small group
  - Activity A (Appendix A): Name the arrhythmia and rate--5 minutes.
  - Activity B (Appendix B): Name the arrhythmia and narrow or wide--5 minutes.
  - Activity C (Appendix C): Name the pulseless rhythm and if it shockable or unshockable--5 minutes.
  - Activity D (Appendix D): Name the H/T and treatment--5 minutes.
    BREAK (5–10 minutes)
6. Review Algorithm Key answers in PowerPoint (Appendices F and H).
7. Case-based learning exercise (Appendix K): 3 cases ~ 30 minutes/ 10 minutes each.
8. Review case-based learning key: (Appendix L).
9. Have students take the post-test individually (10 minutes) (Appendix I).
10. Solicit verbal feedback on workshop.

## Appendix A Activity A: Name the Tachyarrhythmia and Rate

1. Name the tachyarrhythmia and rate (EKG1a)
  ___________________________________________________________________
2. Name the tachyarrhythmia and rate (EKG2a)
  ___________________________________________________________________
3. Name the tachyarrhythmia and rate EKG (EKG3a)
  ___________________________________________________________________
4. Name the tachyarrhythmia and rate (EKG4a)
  ___________________________________________________________________
5. Name the tachyarrhythmia and rate (EKG5a)
  ___________________________________________________________________

## Appendix B Activity B: Narrow or Wide QRS

1. Name the rhythm and is the QRS narrow or wide? (EKG1b)
  ___________________________________________________________________
2. Name the rhythm and is the QRS narrow or wide? (EKG2b)
  ___________________________________________________________________
3. Name the rhythm and is the QRS narrow or wide? (EKG3b)
  ___________________________________________________________________
4. Name the rhythm and is the QRS narrow or wide? (EKG4b)
  ___________________________________________________________________
5. Name the rhythm and is the QRS narrow or wide? (EKG5b)
  ___________________________________________________________________

## Appendix C Activity C: Shockable or Unshockable?

1. Name the rhythm and if it shockable or unshockable (EKG1c)
  ___________________________________________________________________
2. Name the rhythm and if it shockable or unshockable (EKG2c)
  ___________________________________________________________________
3. Name the rhythm and if it shockable or unshockable (EKG3c)
  ___________________________________________________________________
4. Name the rhythm and if it shockable or unshockable (EKG4c)
  ____________________________________________________________________

## Appendix D Activity D: Hs and Ts

1. Name the implicated H or T and how to fix the underlying cause: Patient who has missed dialysis and presents in asystole.
  ____________________________________________________________________
2. Name the implicated H or T and how to fix the underlying cause: Patient who recently had knee surgery and presents in PEA.
  ____________________________________________________________________
3. Name the implicated H or T and how to fix the underlying cause: Patient who has aspiration event at nursing home, initial O2 sat of 70%, and goes into asystole.
  ____________________________________________________________________
4. Name the implicated H or T and how to fix the underlying cause: Patient who had JVD, muffled heart sounds, and hypotension who then goes into PEA.
  ____________________________________________________________________
5. Name the implicated H or T and how to fix the underlying cause: Patient who has +FAST exam in the RUQ following motor vehicle accident and who goes into PEA.
  ____________________________________________________________________

## Appendix I Dangerous Dysrhythmias Pre- & Post-Test

### Use the EKG below to answer questions 1–6. (EKG1b)

1. Identify the rhythm:
  - Atrial fibrillation
  - Supraventricular tachycardia (SVT)
  - Ventricular fibrillation
  - Ventricular tachycardia
  - ST-segment elevation myocardial infarction (STEMI)
2. My confidence in the RECOGNITION of this rhythm is
  **Table:**
  1	2	3	4	5
  Not Confident				Extremely Confident
3. Does the rhythm have a NARROW or WIDE QRS interval?
  - Narrow
  - Wide
4. My confidence in RECOGNITION of a NARROW versus WIDE QRS interval is
  **Table:**
  1	2	3	4	5
  Not Confident				Extremely Confident
5. What CRITICAL ACTION is indicated if the patient is **pulseless** with the above rhythm?
  - Defibrillation
  - Synchronized cardioversion
6. My confidence in the MANAGEMENT of this rhythm is
  **Table:**
  1	2	3	4	5
  Not Confident				Extremely Confident

### Use the EKG below to answer questions 7–12. (EKG3b)

7. Identify the rhythm:
  - Atrial flutter
  - SVT
  - Ventricular tachycardia
  - Ventricular fibrillation
  - STEMI
8. My confidence in the RECOGNITION of this rhythm is
  **Table:**
  1	2	3	4	5
  Not Confident				Extremely Confident
9. Does the rhythm have a NARROW or WIDE QRS interval?
  - Narrow
  - Wide
10. What CRITICAL ACTION is indicated if the patient is UNSTABLE with the above rhythm?
  - Defibrillation
  - Synchronized cardioversion
11. My confidence in the MANAGEMENT of this arrhythmia is
  **Table:**
  1	2	3	4	5
  Not Confident				Extremely Confident
12. Which of the following in a patient with a tachyarrhythmia may indicate that the patient is UNSTABLE?
  - Systolic blood pressure of 100
  - Dry skin
  - Chest pain
  - Glasgow Coma Score= 15
13. My confidence in the RECOGNITION of an UNSTABLE patient with a tachyarrhythmia is
  **Table:**
  1	2	3	4	5
  Not Confident				Extremely Confident
14. What CRITICAL ACTION is indicated in a patient with an UNSTABLE tachyarrhythmia?
  - Defibrillation
  - Synchronized cardioversion
15. My confidence in the MANAGEMENT of a patient with an unstable tachyarrhythmia is
  **Table:**
  1	2	3	4	5
  Not Confident				Extremely Confident
16. Which correctly identifies a dysrhythmia that can be cardioverted OR defibrillated depending on the clinical scenario?
  - Pulseless electrical activity (PEA)
  - Atrial fibrillation
  - Ventricular tachycardia
  - Ventricular fibrillation
17. My confidence in the INDICATION for cardioversion versus defibrillation is
  **Table:**
  1	2	3	4	5
  Not Confident				Extremely Confident
18. Which of the following is an “unshockable” rhythm in cardiac arrest?
  - PEA
  - Ventricular tachycardia
  - Ventricular fibrillation
  - SVT
19. My confidence in the IDENTIFICATION of “shockable” versus “unshockable” rhythms is
  **Table:**
  1	2	3	4	5
  Not Confident				Extremely Confident
20. According to the ACLS algorithm, what treatment is recommended for “un-shockable” rhythms in cardiac arrest?
  - Defibrillation
  - CPR and epinephrine
  - Amiodarone
  - Atropine
21. My confidence in the MANAGEMENT of “shockable” versus “unshockable” rhythms is
  **Table:**
  1	2	3	4	5
  Not Confident				Extremely Confident
22. Which of the following rhythms is ALWAYS pulseless?
  - Ventricular tachycardia
  - SVT
  - PEA
  - Ventricular fibrillation
  - A and D
  - C and D
23. My confidence in the IDENTIFICATION of “pulseless” rhythms is
  **Table:**
  1	2	3	4	5
  Not Confident				Extremely Confident
24. According to the ACLS algorithm, what treatment is recommended for ALL “pulseless” rhythms in cardiac arrest?
  - Defibrillation
  - Amiodarone
  - CPR and epinephrine
  - Atropine
25. My confidence in the MANAGEMENT of “pulseless” rhythms is
  **Table:**
  1	2	3	4	5
  Not Confident				Extremely Confident
26. The most likely cause for a patient presenting in pulseless ventricular tachycardia cardiac arrest is
  - Hypoxia
  - Cardiac ischemia
  - QTc prolongation from medication
  - Trauma
27. My confidence in the IDENTIFYING the cause of pulseless ventricular tachycardia is
  **Table:**
  1	2	3	4	5
  Not Confident				Extremely Confident
28. Which of the following would be the MOST likely cause of a patient presenting in cardiac arrest with an “unshockable” rhythm?
  - Pulmonary embolism
  - STEMI
  - QTc prolongation from medication
  - Hypercalcemia
29. My confidence in IDENTIFYING the cause of “unshockable rhythms” is
  **Table:**
  1	2	3	4	5
  Not Confident				Extremely Confident

## Appendix J Dangerous Dysrhythmias Pre- & Post-Test Key

### Use the EKG below to answer questions 1–6. (EKG1b)

1. Identify the rhythm:
  - Atrial fibrillation
  - Supraventricular tachycardia (SVT)
  - Ventricular fibrillation
  - **Ventricular tachycardia**
  - ST-segment elevation myocardial infarction (STEMI)
2. My confidence in the RECOGNITION of this rhythm is
  **Table:**
  1	2	3	4	5
  Not Confident				Extremely Confident
3. Does the rhythm have a NARROW or WIDE QRS interval?
  - Narrow
  - **Wide**
4. My confidence in RECOGNITION of a NARROW versus WIDE QRS interval is
  **Table:**
  1	2	3	4	5
  Not Confident				Extremely Confident
5. What CRITICAL ACTION is indicated if the patient is **pulseless** with the above rhythm?
  - **Defibrillation**
  - Synchronized cardioversion
6. My confidence in the MANAGEMENT of this rhythm is
  **Table:**
  1	2	3	4	5
  Not Confident				Extremely Confident

### Use the EKG below to answer questions 7–12. (EKG3b)

7. Identify the rhythm:
  - **Atrial flutter**
  - SVT
  - Ventricular tachycardia
  - Ventricular fibrillation
  - STEMI
8. My confidence in the RECOGNITION of this rhythm is
  **Table:**
  1	2	3	4	5
  Not Confident				Extremely Confident
9. Does the rhythm have a NARROW or WIDE QRS interval?
  - **Narrow**
  - Wide
10. What CRITICAL ACTION is indicated if the patient is UNSTABLE with the above rhythm?
  - Defibrillation
  - **Synchronized cardioversion**
11. My confidence in the MANAGEMENT of this arrhythmia is
  **Table:**
  1	2	3	4	5
  Not Confident				Extremely Confident
12. Which of the following in a patient with a tachyarrhythmia may indicate that the patient is UNSTABLE?
  - Systolic blood pressure of 100
  - Dry skin
  - **Chest pain**
  - Glasgow Coma Score= 15
13. My confidence in the RECOGNITION of an UNSTABLE patient with a tachyarrhythmia is
  **Table:**
  1	2	3	4	5
  Not Confident				Extremely Confident
14. What CRITICAL ACTION is indicated in a patient with an UNSTABLE tachyarrhythmia?
  - Defibrillation
  - **Synchronized cardioversion**
15. My confidence in the MANAGEMENT of a patient with an unstable tachyarrhythmia is
  **Table:**
  1	2	3	4	5
  Not Confident				Extremely Confident
16. Which correctly identifies a dysrhythmia that can be cardioverted OR defibrillated depending on the clinical scenario?
  - Pulseless electrical activity (PEA)
  - Atrial fibrillation
  - **Ventricular tachycardia**
  - Ventricular fibrillation
17. My confidence in the INDICATION for cardioversion versus defibrillation is
  **Table:**
  1	2	3	4	5
  Not Confident				Extremely Confident
18. Which of the following is an “unshockable” rhythm in cardiac arrest?
  - **PEA**
  - Ventricular tachycardia
  - Ventricular fibrillation
  - SVT
19. My confidence in the IDENTIFICATION of “shockable” versus “unshockable” rhythms is
  **Table:**
  1	2	3	4	5
  Not Confident				Extremely Confident
20. According to the ACLS algorithm, what treatment is recommended for “unshockable” rhythms in cardiac arrest?
  - Defibrillation
  - **CPR and epinephrine**
  - Amiodarone
  - Atropine
21. My confidence in the MANAGEMENT of “shockable” versus “unshockable” rhythms is
  **Table:**
  1	2	3	4	5
  Not Confident				Extremely Confident
22. Which of the following rhythms is ALWAYS pulseless?
  - Ventricular tachycardia
  - SVT
  - PEA
  - Ventricular fibrillation
  - A and D
  - **C and D**
23. My confidence in the IDENTIFICATION of “pulseless” rhythms is
  **Table:**
  1	2	3	4	5
  Not Confident				Extremely Confident
24. According to the ACLS algorithm, what treatment is recommended for ALL “pulseless” rhythms in cardiac arrest?
  - Defibrillation
  - Amiodarone
  - **CPR and epinephrine**
  - Atropine
25. My confidence in the MANAGEMENT of “pulseless” rhythms is
  **Table:**
  1	2	3	4	5
  Not Confident				Extremely Confident
26. The most likely cause for a patient presenting in pulseless ventricular tachycardia cardiac arrest is
  - Hypoxia
  - **Cardiac ischemia**
  - QTc prolongation from medication
  - Trauma
27. My confidence in the IDENTIFYING the cause of pulseless ventricular tachycardia is
  **Table:**
  1	2	3	4	5
  Not Confident				Extremely Confident
28. Which of the following would be the MOST likely cause of a patient presenting in cardiac arrest with an “unshockable” rhythm?
  - **Pulmonary embolism**
  - STEMI
  - QTc prolongation from medication
  - Hypercalcemia
29. My confidence in IDENTIFYING the cause of “unshockable rhythms” is
  **Table:**
  1	2	3	4	5
  Not Confident				Extremely Confident

## Appendix K Case-Based Learning Exercises

### Case 1

**A 42-year-female presents to the ED with a complaint of palpitations. She has had nausea, vomiting and diarrhea for the last 3 days**.

Triage vitals:

HR (heart rate) 130

BP (blood pressure) 125/80

RR (respiratory rate) 15

O2 (oxygen) sat 98%

Temp (temperature) 99.2° F

A. Circle the abnormal vital sign

Past medical history: None

Social history: Denies tobacco, alcohol and drugs

Surgical history: none

Family history: HTN (hypertension)

The assessment is as follows: A (airway), B (breathing), C (circulation), D (disability)

A: Airway patent

B: RR is 15, no resp. difficulties, lungs CTA (clear to auscultation)

C: BP 125/80, 2+ radial pulses, pulse is tachycardic and irregularly irregular

D: GCS (Glasgow Coma Scale)= 15

SKIN: pale, warm and dry

The following EKG is obtained (EKG2a)

- What is the rhythm and rate?
- Narrow or wide QRS?
- Is the patient currently stable or unstable?
- What labs, imaging tests, medications, and interventions should be considered?
  Labs:
  Imaging:
  Medications:
  Interventions:

1 liter IV fluids are hung. The patient is given 5 mg of Metoprolol for 3 doses timed 5 minutes apart, but she remains in A fib (atrial fibrillation) with RVR (rapid ventricular response) with rate of 130, and BP now 80/60.

A. Is the patient currently stable or unstable?
B. What medications and interventions could be considered?

You are worried that the patient will continue to deteriorate if you do not intervene.

C. Should you perform synchronized cardioversion or defibrillation?

You ask that the tech place pads on the patient in anticipation of emergent cardioversion. You give the patient 50 mcg of fentanyl. The patient is cardioverted with 120 Joules (J). The resultant rhythm is seen below:

(EKG5a)

- What is the rhythm and rate?
- Narrow or wide QRS?
- What is the most likely cause of the dysrhythmia in this patient?
  **Table:**
  Na	142
  K	2.8
  Cl	100
  BUN	40
  Cr	2.0
  Glucose	99
  Bicarb	25
  TSH, troponin	Within Normal Limits
- What is the anion gap?
- What medications and interventions should be considered?

### Case 2

**A 65-year-old male presents to the ED with a complaint of chest pain**.

Triage vitals:

HR 60

BP 90/50

RR 15

O2 sat 98%

Temp 98.6° F

A. Circle the abnormal vital sign.

A: Airway patent

B: RR is 15, no resp. difficulties, lungs CTA

C: BP 90/50, 2+ radial pulses, pulse is regular

D: GCS= 15

Skin: Diaphoretic

Past medical history: DM (diabetes mellitus), HTN (hypertension)

Social history: Ex smoker (1 ppd for 30 years), occasional EtOH (alcohol use), denies drug use

Surgical history: cholecystectomy, appendectomy

Family history: HTN

The following EKG was obtained from triage (EKG5b)

- What is the rhythm?
- Is the patient stable or unstable?
- What medications and interventions should be considered right now?

You recognize that the EKG shows an inferior STEMI (ST elevation myocardial infarction) and based on the patient’s hypotension, you are concerned regarding right ventricle infarction.

You give the patient 324 mg of Aspirin, 1 liter of IV fluids and a bolus of IV heparin while you page cardiology. You avoid nitroglycerin which will worsen the hypotension.

You are waiting for the cardiologist to call you back when the nurse alerts you to a change on the telemetry monitor (EKG5c)

- What is the rhythm and rate?
- Is this rhythm narrow or wide?

You race to the patient’s bedside and repeat the ABCDs.

A: not responding

B: no spontaneous respirations

C: no palpable peripheral pulse

D: GCS 3

- What medications and interventions should be considered right now?
  Medications:
  Interventions:
- Does the patient need synchronized cardioversion or defibrillation?
- What is the most likely cause of the dysrhythmia in this patient?

The patient receives 3 rounds of CPR (cardiopulmonary resuscitation), 1 mg epinephrine x 3, defibrillation x 3, and 300 mg of IV Amiodarone. ROSC (return of spontaneous circulation) is achieved, and the patient is taken emergently to the cath lab where he was found to have 90% blockage of the right coronary artery with involvement of the posterior wall.

### Case 3

**A 45-year-old male brought by police for “medical clearance” for jail**.

Vitals:

HR 150

BP 190/100

RR 15

O2 sat 98%

Temp 99° F

A. Circle the abnormal vital signs

A: Airway patent

B: RR is 15, no resp. difficulties, lungs CTA

C: BP 190/100, 2+ radial pulses, pulse is regular

D: GCS= 15

Skin: Diaphoretic

Past medical history: None

Social history: Admits to cocaine use prior to arrival

Surgical history: Wisdom tooth extraction

Family history: HTN, hyperthyroidism

(EKG5d)

- What is the rhythm?
- Is the rhythm narrow or wide?
- Is the patient stable or unstable?
- What medications and interventions should be considered right now?
  Medications:
  Interventions:

You recognize that the EKG shows SVT (supraventricular tachycardia). You hang 1 liter of IV fluids, give 1 mg of Ativan for agitation, and decide to give Adenosine to chemically cardiovert the patient. You give 3 doses; 6 mg x 2, then 12 mg, but are unsuccessful.

Since the patient is currently stable, you are considering using the 2nd line agent diltiazem when the nurse alerts you that the patient’s blood pressure has dropped.

You race to the patient’s bedside and repeat the ABCDs.

A: not responding

B: RR 12, bilateral breath sounds present

C: HR 150, BP 80/50

D: GCS 14 (Eyes=4, Verbal=4, Motor=6)

Skin: diaphoretic, pale

- Is the patient stable or unstable?
- What medications and interventions should be considered right now?
- What is the most likely cause of the dysrhythmia in this patient?

You decide to perform synchronized cardioversion with 120 J and the rhythm strip shows the resultant rhythm.

(EKG2c)

- What is the rhythm and rate?
- Is the rhythm narrow or wide?
- What medications and interventions should be considered right now?
  Medications:
  Interventions:

Since the patient was just cardioverted, the physician turns the Zoll to the defibrillator setting. After one 200 J defibrillation, the patient regains ROSC. The patient is in turn admitted overnight to the hospital for observation.

## Appendix L Case-Based Learning Exercises Key

### Case 1

Atrial fibrillation, stable from hypokalemia, then becomes unstable requiring cardioversion

### Case 2

Inferior STEMI, that then becomes pulseless V tach, requiring defibrillation

### Case 3

SVT that undergoes synchronized cardioversion into V fib, requiring defibrillation

### Case 1

**A 42-year-female presents to the ED with a complaint of palpitations. She has had nausea, vomiting and diarrhea for the last 3 days**.

Triage vitals:

*HR (heart rate) 130*

BP (blood pressure) 125/80

RR (respiratory rate) 15

O2 (oxygen) sat 98%

Temp (temperature) 99.2° F

A. Circle the abnormal vital sign

Past medical history: None

Social history: Denies tobacco, alcohol and drugs

Surgical history: none

Family history: HTN (hypertension)

The assessment is as follows: A (airway), B (breathing), C (circulation), D (disability)

A: Airway patent

B: RR is 15, no resp. difficulties, lungs CTA (clear to auscultation)

C: BP 125/80, 2+ radial pulses, pulse is tachycardic and irregularly irregular

D: GCS (Glasgow Coma Scale)= 15

SKIN: pale, warm and dry

The following EKG is obtained (EKG2a)

- What is the rhythm and rate?
  *Atrial fibrillation with RVR rate 132*
- Narrow or wide QRS?
  *Narrow*
- Is the patient currently stable or unstable?
  *Stable-mentating well, SBP (systolic blood pressure) >90*
- What labs, imaging tests, medications, and interventions should be considered?
  Labs:
    *CBC rule out anemia, CMP-check K and renal function, BNP depending on fluid status, troponin, Magnesium, TSH. Infectious workup-does she need CT abdomen pelvis to rule out colitis given diarrhea?*
  Imaging:
    *CXR -assess for signs of heart failure*
  Medications:
    *Diltiazem or Metoprolol for rate control, IV fluids*
  Interventions:
    *None*

1 liter IV fluids are hung. The patient is given 5 mg of Metoprolol for 3 doses timed 5 minutes apart, but she remains in A fib (atrial fibrillation) with RVR (rapid ventricular response) with rate of 130, and BP now 80/60.

A. Is the patient currently stable or unstable?
  *Unstable because of hypotension*
What medications and interventions could be considered?
  *Consider synchronized cardioversion*

You are worried that the patient will continue to deteriorate if you do not intervene.

C. Should you perform synchronized cardioversion or defibrillation?
  *Synchronized cardioversion*

You ask that the tech place pads on the patient in anticipation of emergent cardioversion. You give the patient 50 mcg of fentanyl. The patient is cardioverted with 120 Joules (J). The resultant rhythm is seen below:

(EKG5a)

- What is the rhythm and rate?
  *Atrial flutter with 4:1 conduction, rate of 65*
- Narrow or wide QRS?
  *Narrow*
- What is the most likely cause of the dysrhythmia in this patient?
  *Hypokalemia*
  **Table:**
  Na	142
  K	2.8
  Cl	100
  BUN	40
  Cr	2.0
  Glucose	99
  Bicarb	25
  TSH, troponin	Within Normal Limits
- What is the anion gap?
  *Na - ( Cl+ HCO3)*
  *142- (110+25)*
  *142–135= 7*
- What medications and interventions should be considered?
  *IV fluids for AKI*
  *Potassium repletion: IV K, oral K and IV Magnesium*

### Case 2

**A 65-year-old male presents to the ED with a complaint of chest pain**.

Triage vitals:

HR 60

*BP 90/50*

RR 15

O2 sat 98%

Temp 98.6° F

A. Circle the abnormal vital sign.

A: Airway patent

B: RR is 15, no resp. difficulties, lungs CTA

C: BP 90/50, 2+ radial pulses, pulse is regular

D: GCS= 15

Skin: Diaphoretic

Past medical history: DM (diabetes mellitus), HTN (hypertension)

Social history: Ex smoker (1 ppd for 30 years), occasional EtOH (alcohol use), denies drug use

Surgical history: cholecystectomy, appendectomy

Family history: HTN

The following EKG was obtained from triage (EKG5b)

- What is the rhythm?
  *Inferior STEMI*
- Is the patient stable or unstable?
  *Unstable -SBP 90, diaphoretic*
- What medications and interventions should be considered right now?
  *Medications: aspirin (ASA) 324 mg, avoid nitroglycerin (NTG), heparin bolus, fluids Interventions: activate STEMI alert for emergent cardiac catheterization, place defib pads on patient in case of deterioration*

You recognize that the EKG shows an inferior STEMI (ST elevation myocardial infarction) and based on the patient’s hypotension, you are concerned regarding right ventricle infarction.

You give the patient 324 mg of Aspirin, 1 liter of IV fluids and a bolus of IV heparin while you page cardiology. You avoid nitroglycerin which will worsen the hypotension.

You are waiting for the cardiologist to call you back when the nurse alerts you to a change on the telemetry monitor (EKG5c)

- What is the rhythm and rate?
  *Ventricular tachycardia, rate ~300*
- Is this rhythm narrow or wide?
  *Wide*

You race to the patient’s bedside and repeat the ABCDs.

A: not responding

B: no spontaneous respirations

C: no palpable peripheral pulse

D: GCS 3

- What medications and interventions should be considered right now?
  Medications:
    *Epinephrine, Amiodarone, tPA*
  Interventions:
    *CPR, Defibrillation*
- Does the patient need synchronized cardioversion or defibrillation?
  *Defibrillation*
- What is the most likely cause of the dysrhythmia in this patient?
  *Cardiac ischemia*

The patient receives 3 rounds of CPR (cardiopulmonary resuscitation), 1 mg epinephrine x 3, defibrillation x 3, and 300 mg of IV Amiodarone. ROSC (return of spontaneous circulation) is achieved, and the patient is taken emergently to the cath lab where he was found to have 90% blockage of the right coronary artery with involvement of the posterior wall.

### Case 3

**A 45-year-old male brought by police for “medical clearance” for jail**.

Vitals:

*HR 150*

*BP 190/100*

RR 15

O2 sat 98%

Temp 99° F

A. Circle the abnormal vital signs

A: Airway patent

B: RR is 15, no resp. difficulties, lungs CTA

C: BP 190/100, 2+ radial pulses, pulse is regular

D: GCS= 15

Skin: Diaphoretic

Past medical history: None

Social history: Admits to cocaine use prior to arrival

Surgical history: Wisdom tooth extraction

Family history: HTN, hyperthyroidism

(EKG5d)

- What is the rhythm?
  *SVT*
- Is the rhythm narrow or wide?
  *Narrow*
- Is the patient stable or unstable?
  *Stable*
- What medications and interventions should be considered right now?
  Medications:
    *Adenosine, avoid beta blockers with cocaine!*
  Interventions:
    *Vagal maneuvers, Ativan*

You recognize that the EKG shows SVT (supraventricular tachycardia). You hang 1 liter of IV fluids, give 1 mg of Ativan for agitation, and decide to give Adenosine to chemically cardiovert the patient. You give 3 doses; 6 mg x 2, then 12 mg, but are unsuccessful.

Since the patient is currently stable, you are considering using the 2nd line agent diltiazem when the nurse alerts you that the patient’s blood pressure has dropped.

You race to the patient’s bedside and repeat the ABCDs.

A: not responding

B: RR 12, bilateral breath sounds present

C: HR 150, BP 80/50

D: GCS 14 (Eyes=4, Verbal=4, Motor=6)

Skin: diaphoretic, pale

- Is the patient stable or unstable?
  *Unstable-now hypotensive*
- What medications and interventions should be considered right now?
  *Synchronized cardioversion*
- What is the most likely cause of the dysrhythmia in this patient?
  *Cocaine*

You decide to perform synchronized cardioversion with 120 J and the rhythm strip shows the resultant rhythm.

(EKG2c)

- What is the rhythm and rate?
  *Ventricular fibrillation*
- Is the rhythm narrow or wide?
  *Wide*
- What medications and interventions should be considered right now?
  Medications:
    *Epinephrine, Amiodarone*
  Interventions:
    *CPR, defibrillation*

Since the patient was just cardioverted, the physician turns the Zoll to the defibrillator setting. After one 200 J defibrillation, the patient regains ROSC. The patient is in turn admitted overnight to the hospital for observation.
