# Supplementary material for: Dangerous Dysrhythmias in the ED: An Interactive EKG Workshop for Emergency Medicine Clerkship Learners
Source: J Educ Teach Emerg Med. 2026 Jul 31;11(3):SG1–SG68. doi: 10.5070/M5.52195 (PMC13441274; doi:10.5070/M5.52195)
Supplement: Supplementary file 1 [file 11-3-SG1-Appendix_E.pdf]

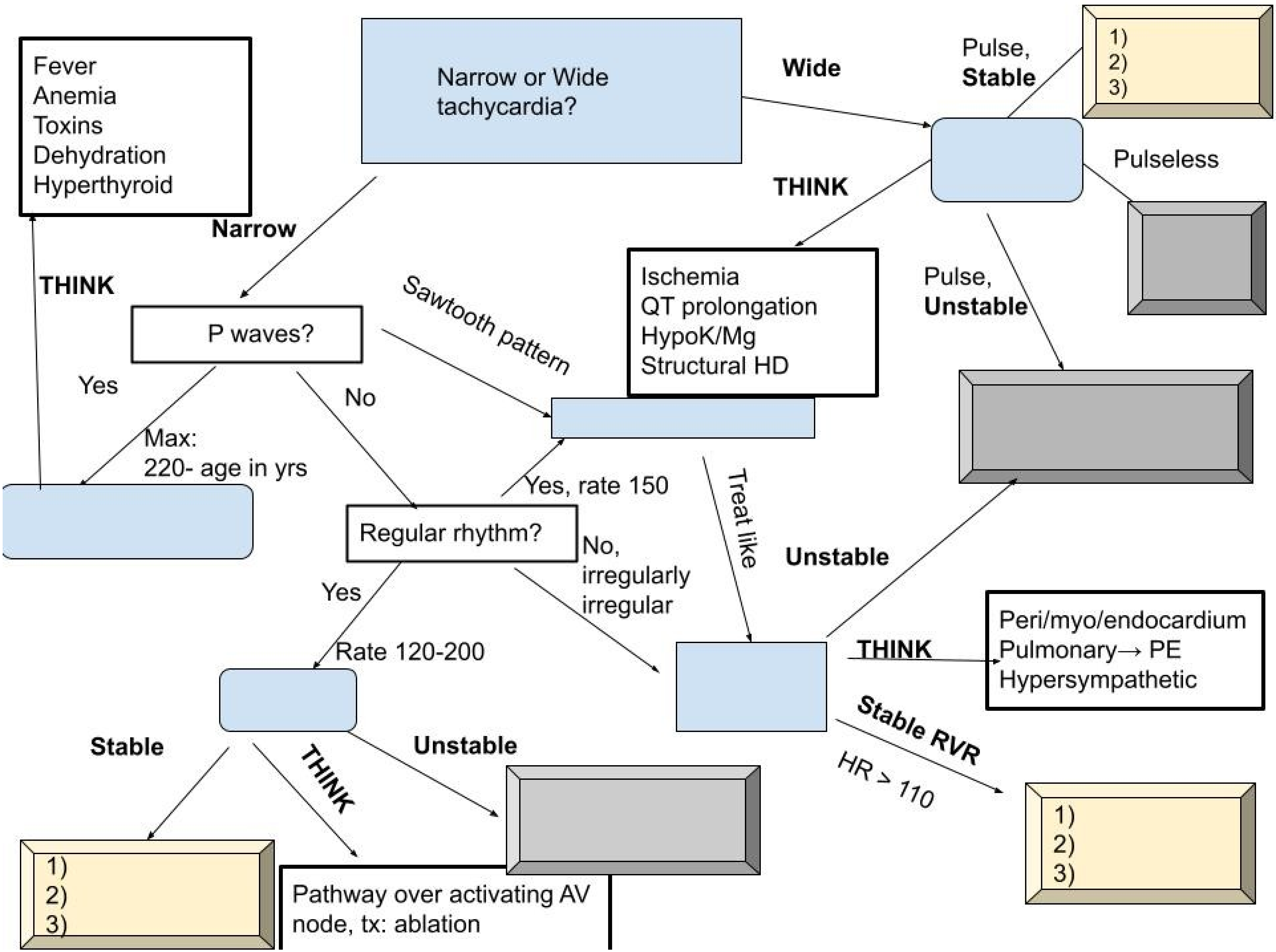
Narrow or Wide tachycardia?
Wide
Pulse, Stable
1)
2)
3)
Pulseless
THINK
Ischemia
QT prolongation
HypoK/Mg
Structural HD
Pulse, Unstable
Fever
Anemia
Toxins
Dehydration
Hyperthyroid
Narrow
THINK
P waves?
Sawtooth pattern
Yes
No
Max:
220- age in yrs
Yes, rate 150
Treat like
Regular rhythm?
No, irregularly irregular
Unstable
Yes
Rate 120-200
THINK
Peri/myo/endocardium
Pulmonary→ PE
Hypersympathetic
Stable
THINK
Unstable
Stable RVR
HR > 110
1)
2)
3)
1)
2)
3)
Pathway over activating AV node, tx: ablation
