# Supplementary material for: Dangerous Dysrhythmias in the ED: An Interactive EKG Workshop for Emergency Medicine Clerkship Learners
Source: J Educ Teach Emerg Med. 2026 Jul 31;11(3):SG1–SG68. doi: 10.5070/M5.52195 (PMC13441274; doi:10.5070/M5.52195)

Unstable:

1.
2.
3.
4.
5.

Approach:

A:

B:

C:

D:

E:

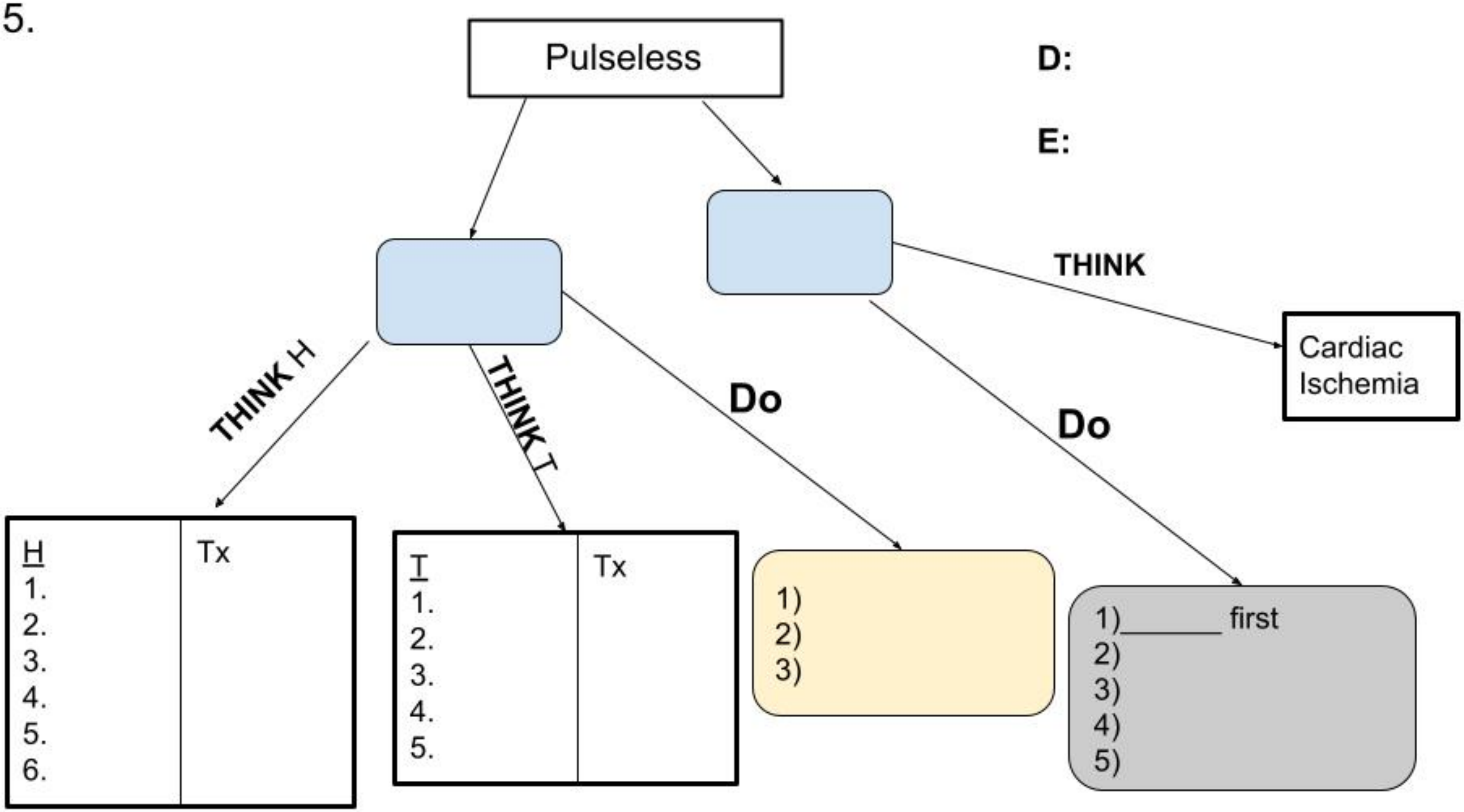

Supplement: Supplementary file 3 [file 11-3-SG1-Appendix_G.pdf]
